# A Biomechanical Analysis of Two-Person Emergency Patient Lifting Techniques Using Motion Capture and Ergonomic Assessment

**DOI:** 10.3390/s26092747

**Published:** 2026-04-29

**Authors:** Xiaoxu Ji, Xin Gao, Paige L. Johnson, Isaac Wheeler

**Affiliations:** 1 Biomedical Engineering, Gannon University, Erie, PA 16541, USA; johnson231@gannon.edu (P.L.J.); wheeler039@gannon.edu (I.W.); 2 Electrical and Computer Engineering, Gannon University, Erie, PA 16541, USA; gao005@gannon.edu

**Keywords:** emergency responders, rescue, carrying techniques, musculoskeletal disorders, motion capture, ergonomics

## Abstract

Emergency responders face a high risk of musculoskeletal disorders (MSDs), particularly lower back injuries, due to frequent patient-handling tasks performed in awkward and dynamic postures. This aim of study is to utilize dual motion capture systems integrated with a digital human modeling (DHM) ergonomics tool to evaluate the biomechanical effects of two common two-person carrying techniques: facing forward and facing each other. Twenty-two participants lifted a 25 kg mannequin while wearing Xsens motion sensors, and lumbar forces and joint angles were analyzed using Siemens Jack software (v9.0). Peak compressive and anterior–posterior (AP) shear forces, along with trunk, hip, and knee joint angles, were examined. Compressive forces ranged from approximately 948.6 to 2955.6 N, and AP shear forces ranged from 286.0 to 827.0 N. Mean compressive and AP shear forces were higher during the facing-each-other task (1977.3 N and 595.0 N) than during the facing-forward task (1596.0 N and 462.0 N). Males experienced higher spinal loads than females across both tasks. The facing-each-other technique was associated with greater hip flexion, lower knee flexion, and reduced trunk flexion, whereas the facing-forward technique resulted in less hip flexion, greater knee flexion, and greater trunk flexion. Overall, under the conditions of the present study, the facing-forward technique was associated with lower lumbar loading indicators. Integrating motion capture with DHM offers a valuable approach for evaluating realistic rescue tasks and can inform ergonomic training strategies for emergency responders.

## 1. Introduction

First responders, such as firefighters, paramedics, and emergency medical technicians (EMTs), play a crucial role in ensuring a patient’s well-being before they reach the emergency department. However, 62% of the back injuries received by prehospital care providers are caused by lifting the patient, resulting in an increased risk of MSDs [1]. Within recent years, sprains and strains on the upper trunk and low back were the most common injury among paramedics, EMTs, and firefighters [2,3,4]. In fact, within the first four years of joining the occupation, almost 25% of emergency medical service (EMS) providers who developed a back injury were forced to end their career [5]. Additionally, the most common injury related to the early retirement of a firefighter is to the lower back [6]. Repetitive forceful movements along with twisting and bending while lifting loads are among the most influential causes of musculoskeletal disorders [7]. Increased compressive, shear, and torque forces on the spine during a change in posture elevate MSDs. The force imposed on the lumbar vertebrae by the patient’s weight while lifting, along with the posture adopted by an EMS employee, are two of the major factors that increase the risk of injury. Although the Occupational Safety and Health Administration set restrictions on the load limit to professionals in healthcare, there has been a relatively small decrease in back injuries [8]. Currently, there is a shortage of EMS personnel [9,10]. With increasing obesity rates across the United States and an estimated need for 40,000 additional staff members by 2030, proper lifting precautions are more important now than ever [11,12].

Methods of assessing and reducing injury risk while lifting often involve the use of surveys and patient lifting equipment [3,13]. Previous research has also explored modified lifting ergonomics by reducing friction and adjusting the lift height [14]. While surveys may result in the addition of ergonomics training, they do not provide a quantifiable evaluation of injury risk. Additionally, lifting equipment may aid in reducing injury, but it is not practical in particular rescue scenarios. The addition of simulations and technological tools can offer a more precise evaluation of human biomechanics, particularly when two EMS personnel are lifting a patient.

Prior studies have utilized simulations, real-time motion tracking, and muscle activation technology to assess changes in lumbar force, joint angles, and effort exerted while lifting. A DHM software called OpenSim studied the lumbar spine during varying weights for lifting tasks to evaluate the load impact on the spine [15]. Jack, an alternative DHM technology, has been used to evaluate the biomechanics of patient lifting [16]. Posture testing using Jack software has also been heavily explored. The results of DHM in healthcare ergonomics have demonstrated improved ways of performing tasks involving transferring patients laterally. This software has also been used in conjunction with the real-time motion technology, Xsens, to assess the risk of musculoskeletal injury for workers in the healthcare industry [17]. Furthermore, Xsens has been used in combination with electromyography (EMG) to identify the relationship between joint angle changes and muscle activation during lifting [18]. However, each technology presents with shortcomings when used independently. DHM simulations run under ideal conditions and fail to consider the natural variability in human movement during the execution of a lift. Furthermore, while Xsens has been used to evaluate multiple subjects, the technology cannot analyze the change in L4/L5 compression force which assists in indicating injury risk. Additionally, the sensors have not assessed how one’s individual posture influences another during a two-person lift. Moreover, EMGs can track force variations but they are limited to measuring activity in superficial muscles.

Recent advances in motion analysis have also incorporated high-precision imaging techniques, such as the combination of motion capture with dual fluoroscopy, to quantify in vivo joint kinematics with high accuracy [19]. While these approaches provide detailed insights into skeletal motion, they are typically limited to controlled laboratory or clinical environments and are less suitable for dynamic, multi-subject tasks such as team-based patient handling.

A majority of DHM, Xsens, and EMG studies focus on a singular subject during lifting. Although current studies address different lifting techniques and preventative measures to lower injury risk, there is a demand for a more accurate evaluation of human biomechanics while two people are lifting a load. Being that lifting and positioning are among the leading causes of back injury, there is a need for more research that mimics the common lifts among EMTs. EMTs follow guidelines requiring two or more people to be involved in the lift of a patient. The comparison of lifting techniques involving more than one individual, specifically lifting from the ground, has not been heavily researched. The introduction of two people to the integration of real-time motion-tracking technology with DHM is essential to research accuracy, as it enables precise analysis of lifting positions to determine the safest and most efficient approach in an emergency situation. This research will target the specific lifting approaches that are the least likely to result in a spinal injury while rescuing a patient.

In addition to traditional ergonomic and biomechanical approaches, recent studies have explored the application of robotic and assistive technologies to reduce physical workload and improve movement precision. For example, advanced control strategies have been developed for human–robot interaction to achieve accurate torque estimation and motion tracking in lower-limb rehabilitation systems [20]. Similarly, trajectory planning methods have been proposed to enhance movement efficiency and safety in robotic-assisted procedures, including fracture reduction and minimally invasive surgery [21]. These approaches highlight the growing role of robotics in supporting human movement and reducing biomechanical strain. However, such systems are typically designed for controlled clinical or rehabilitation environments and are not directly applicable to dynamic, team-based patient-handling scenarios in EMS settings. Therefore, the present study focuses on human-centered biomechanical analysis using motion capture and digital human modeling to evaluate injury risk during two-person lifting tasks.

The proposed research will employ the real-time motion of two people using Xsens (Movella, Enschede, The Netherlands), alongside Jack software to analyze the effects of patient lifting techniques on spinal forces. This research aims to bridge the gap in understanding how postures influence each other while two people perform a lift while simultaneously utilizing two motion capture systems. Xsens uses sensors that are based on the orientation of an individual to monitor joint angle changes during a movement. The motion capture technology will be synchronized with the DHM simulation, Jack Siemens, to enhance the biomechanics of EMS personnel while lifting a patient during a rescue scenario. By combining motion capture technology with digital human modeling, the effect of changing joint angles on lumbar forces can be represented. A precise assessment of the varied forces in the lower back will be produced by Jack. The integration of technology to simultaneously assess two individuals will reduce the limitations of previous studies. The analysis of human-generated joint angles and lumbar spinal forces will allow key variables to be identified. This could aid in reducing future injuries to EMS workers. The objective of this study is to use the combination of Xsens and Jack to evaluate injury risk of the spine of emergency medical technicians while lifting a patient.

## 2. Methods

### 2.1. Participants and Software

A total of 22 participants participated in the study, 8 of which were male and 14 of which were female. The sample size was calculated after a priori power calculation, based on setting input parameters with a *t*-test, tails (1), effect size (0.6), an error (0.05), and power (0.8). Anthropometric data was recorded from each of the participants to ensure accurate digital models. Measurements included shoulder and hip width, arm span, and upper and lower limb length in addition to height and weight. Males presented with an average height and weight of 175.8 ± 8.3 cm and 77.4 ± 17.7 kg while females displayed an average height and weight of 162.2 ± 6.0 cm and 60.8 ± 4.0 kg. The foot length of each subject was also recorded to certify proper calibration of Xsens and precise detection of joint angle movements. In this study, one fixed participant (S0), with a height of 175 cm and a body weight of 74 kg, was paired with 22 participants to complete the designated tasks. The rationale was that multiple interacting factors, including anthropometrics, posture, lifting technique, and load distribution, can significantly influence lower back forces. This study used a fixed participant with approximately 50th percentile body height and weight, who was fully trained by the Physical Therapy program prior to data collection, enabling one side of the interaction to be standardized while systematically varying the other across 22 participants. The inclusion of 22 unique pairings increases the diversity of interaction scenarios and substantially reduces the likelihood of missing critical combinations of influencing factors. During each data collection session, the fixed participant also instructed participants on proper body mechanics to help minimize the risk of injury.

The motion capture system, Xsens Awinda, was used to collect and record data from the 22 participants during each task of the study [22]. Two full sets of motion systems were required to record the full body movement. Xsens is a wireless real-time motion-tracking system comprising 17 inertia-based sensors. Each sensor corresponds to a specific location on the body chosen to avoid interference from muscle contraction. The sensors are secured to the participant’s head, sternum, shoulders, upper limbs, hands, pelvis, lower limbs, and feet using straps which are positioned around each body segment. The orientation and reference plane must be set following calibration of the sensors since they are based on inertial measurement units (IMUs). The IMUs are made up of 3D linear accelerometers, magnetometers, and gyroscopes to ensure motion can be tracked based on the participant’s orientation in the absence of cameras. A Kalman Filter is employed in each sensor to compute the location of each device and prevent drifts after the initial calibration. The filter integrates inertial data with magnetometer data to effectively estimate the three-dimensional orientation. This feature is essential to the evaluation of lifting practices since minimal joint angle changes can vary the corresponding compressive or shear force exerted on the L4/L5 and L5/S1 vertebrae [23,24]. MVN Analyze software (Movella, Enschede, The Netherlands) simultaneously measures two subjects and the recorded data can be used to create DHMs of each participant.

**Figure 1:**
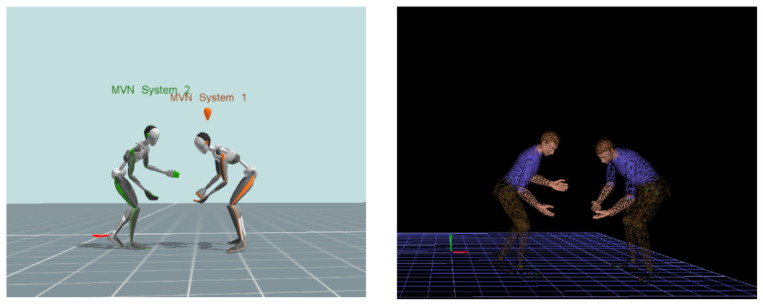
Synchronous movements from Xsens (**left**) and Jack software (**right**).

Siemens Jack is a simulation tool which enables the user to create digital human models for ergonomic evaluation. The software was utilized to create DHMs used for injury risk analysis during each lift based off the anthropometric data provided by each participant. The use of two DHMs provides an accurate biomechanical analysis of the tasks performed ensuring human variability is included in the evaluation of forces imposed on each participant. To aid in the accuracy of the investigation, a port number was used within the network streamer to synchronize the model within Xsens with the created DHMs, as shown in Figure 1. Prior to the beginning of each trial, participants defaulted to a reference position. Each individual stood facing forward with their feet shoulder-width apart and their hands at their sides. This ensured that as data was crossing the port, it was transmitted with accuracy.

### 2.2. Operational Tasks

The investigation consisted of two tasks. Every task was repeated three times. Prior to each task, participants received a demonstration of proper lifting mechanics. They were provided the opportunity to practice the demonstrated techniques before data collection began. During each trial, the participant, alongside a research assistant (S0), lifted and carried a 25 kg mannequin based on ethical considerations. At the start of every trial, the mannequin was positioned on the floor in the supine position. Between tasks, participants were allowed to rest before proceeding to the next lift. The tasks and trials are provided below.

**Figure 2:**
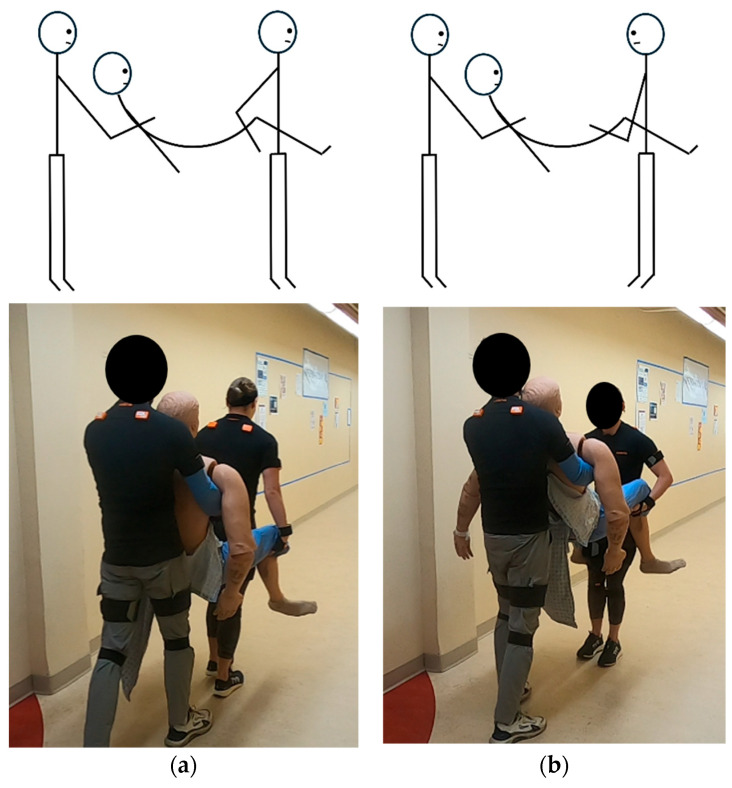
Each lifting technique is demonstrated by the participants: (**a**) two-person fore-and-aft carry while both participants face forward, (**b**) two-person fore-and-aft carry while both participants face the mannequin.

Task 1, facing forward: Two-person fore-and-aft carry while both participants face forward on the floor, as shown in Figure 2a.

In this task, the participant in front placed their hands under the mannequin’s legs and the participant (S0) in the back secured their grasp on the mannequin’s forearms by reaching under its armpits. The participant in the back counted to three so the two participants would lift the mannequin at the same time ensuring the forces they exerted coincided. Following the lift, they carry the mannequin forward 3 m, stop, then place the mannequin on the floor returning it to the supine position. Because the fixed participant (S0) performed the task with 22 different partners, the dataset consisted of 22 distinct subject pairs.

Task 2, facing each other: Two-person fore-and-aft carry while both participants face the mannequin on the floor, as shown in Figure 2b.

This task is identical to Task 1 with an exception; both participants faced the mannequin as opposed to looking forward. The lift was then executed. The mannequin was carried forward 3 m and placed on the floor returning it to the supine position.

### 2.3. Data Analysis

An analysis of each task was performed utilizing both Jack and Xsens to ensure the natural variability of human motion was accounted for. While Xsens provided Jack with an accurate guide to the continuous motion, the force exerted by each hand while lifting was established with a digital force gauge (SF-500) (Wenzhou, China). The applied force depended on both participants’ positions. This force was incorporated into each DHM simulation and for each task using the Force Solver within Jack (Siemens PLM software, v9.0). Hand loads varied slightly across individuals, tasks, and repetitions at the specific posture analyzed. However, these variations were minimal due to the constant mannequin mass. A hand force of 182 N was applied to S0, and 68 N to the participants. Although a constant hand force was applied in the simulation, the selected force value was derived from dynamic measurements during the lifting task, which inherently captured the effects of both tilting angle and vertical acceleration throughout the movement. Importantly, the maximum forces were recorded at the instant when the mannequin was lifted from a stationary position, a phase characterized by transient imbalance and peak acceleration. Additionally, the Jack Siemens ergonomics tool (Siemens PLM software, v9.0) was used to estimate lower back forces in real time, incorporating applied hand forces and adopted postures. The peak lower back forces consistently occurred at the moment when both participants initiated the lift from the floor. This approach was intentionally chosen to represent a worst-case loading condition at lift-off, as this phase poses the greatest risk of injury to the participants due to the combined effects of acceleration, load shift, and body posture.

DHMs within Jack were used to evaluate an approximation of the compressive and AP shear forces exerted on the L4/L5 vertebrae. The lumbar spine was identified as the center of interest for this study. Lower vertebrae, specifically the L4/L5 and L5/S1, experience the highest risk of injury and most extensive stress concentration during lifting [25]. This is because the lumbar spine is responsible for a majority of the support provided to the body. It allocates stability to the cervical and thoracic spine and serves as a point of attachment for numerous muscles [23]. The compressive force on the L4/L5 vertebrae varies throughout the lift due to the changing alignment of the spine. The highest risk of injury was defined as the point of maximum compression force because of this. Individuals face a higher risk of injury to their lumbar spine when the compressive force at the L4/L5 level exceeds 3400 N [26] and the shear force at L5/S1 surpasses 700 N [27]. There is evidence to suggest an individual can obtain an injury to their lower back prior to reaching these limits, especially during repetitive loading, emphasizing the need to analyze injury risk [24]. The analysis provided by Jack can be applied to future patient lifting practices and can predict the lift that imposes the lowest injury risk to rescue personnel.

In addition to the analysis of spinal forces, specific joint angles were selected for evaluation. Every task involved the assessment of the same joint angles: flexion/extension of the trunk, hips, and each knee. The measurements were sourced from both Xsens and Jack. The lower body joints were the focal point due to their significant role in maintaining posture during lifting. This emphasis reduced the reliance of the upper body extremities on the forces generated by the spine. The resulting angles can aid in understanding how the force was distributed throughout the spine.

### 2.4. Statistical Analysis

An analysis was conducted to examine differences between tasks and genders, as well as the relationships among key variables. The Kolmogorov–Smirnov test was performed to assess normality. Additionally, cross-correlation values were calculated to evaluate how body height, joint angles, compressive force, and shear force contribute to injury risk in the L4/L5 region. A linear mixed-effect model, 95% confidence intervals, *p*-values and effect sizes across tasks and between genders were also analyzed. Lifting task (two levels) and gender were included as fixed effects, along with their interaction, while subject was treated as a random effect. For each dependent variable (lower back forces and joint angles), the three repetitions per task were averaged for each participant, and the resulting mean values were used in the analysis. The statistical analysis aimed to identify the most influential variables within each task and to determine lifting techniques that minimize lower back injury risk.

## 3. Results

### 3.1. Lower Back Forces

In this study, the movements in both tasks were symmetrical. Therefore, although lateral shear forces were measured, they were minimal and can be considered negligible.

When facing each other, the compressive forces present in the subject ranged from 1237.8 N to 2955.6 N while the AP shear forces ranged from 407.4 N to 827.0 N. In the facing-forward task, compressive forces ranged from 948.6 N to 2449.5 N, while AP shear forces were lower, between 286.0 N and 654.5 N.

**Figure 3:**
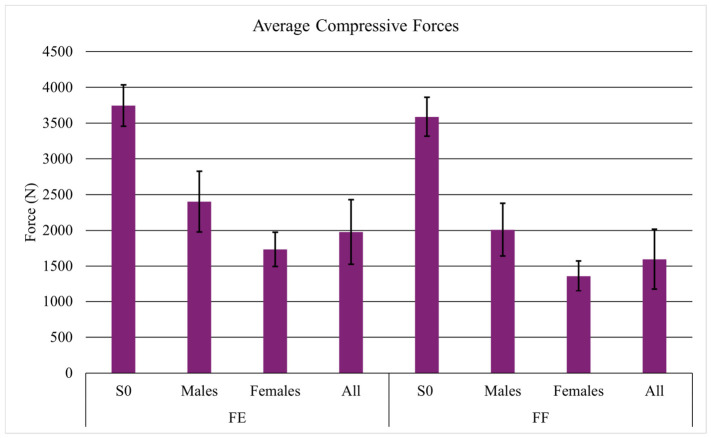
Average compressive forces with standard deviation for S0, males, females, and all participants. FE stands for the facing-each-other tasks, and FF stands for the facing-forward task.

**Figure 4:**
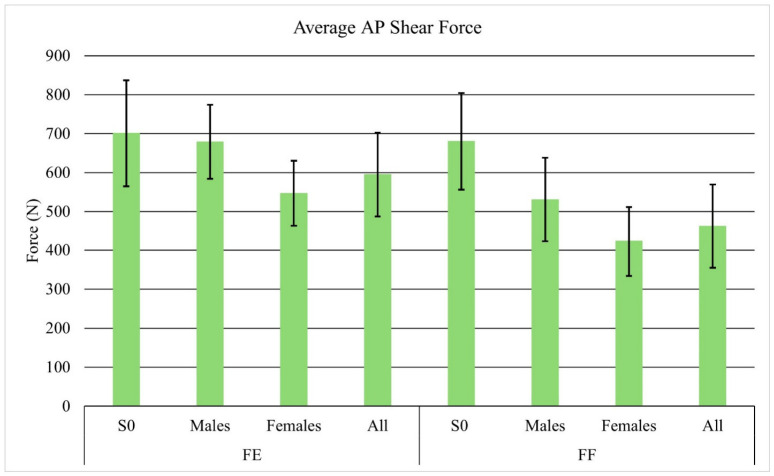
Average AP shear forces with standard deviation for S0, males, females, and all participants.

The average compressive forces for the subjects and S0 during the facing-each-other task were 1977.3 N and 3744.2 N (Figure 3), respectively, while during the facing-forward task they were 1596.3 N and 3587.9 N (Figure 3). Similarly, the average AP shear forces were 595.0. N and 700.8 N when facing each other (Figure 4), and 462.0 N and 680.3 N when facing forward (Figure 4).

On average, male subjects experienced a higher compressive force and AP shear force during both tasks. When facing each other, the average compressive forces for male and female subjects were 2402.4 N vs. 1734.4 N while average AP shear forces were 679.1 N vs. 546.9 N. When facing forward, the average compressive forces for male and female subjects were 2009.6 vs. 1360 N while average AP shear forces were 530.5 N vs. 422.8 N.

### 3.2. Segment Joints

In the facing-each-other task, hip joint angles ranged from 77.6° to 130.9°, knee joint angles from 24.5° to 124.5°, and trunk joint angles from 2.7° to 46.4°, respectively. When facing forward, these ranges shifted from 69.6° to 141.9° for the hip, 36° to 128.3° for the knee, and 11.2° to 44.9° for the trunk.

**Figure 5:**
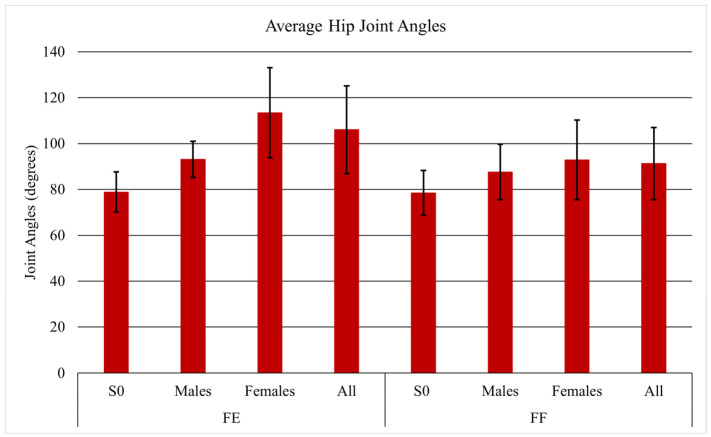
Average hip joint angles with standard deviation for S0, males, females, and all participants.

**Figure 6:**
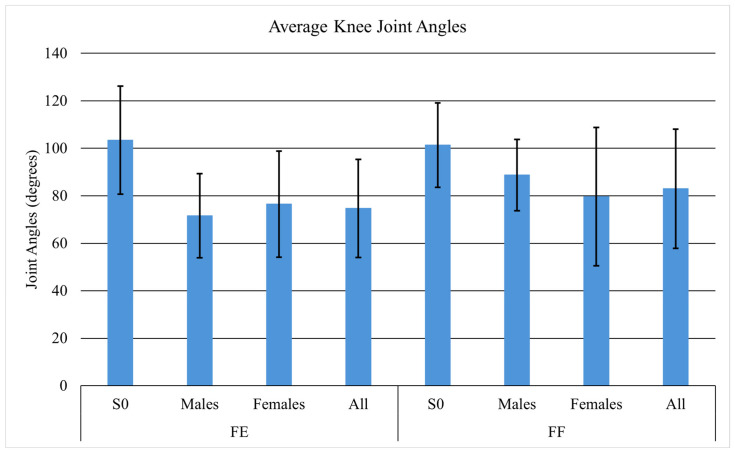
Average knee joint angles with standard deviation for S0, males, females, and all participants.

**Figure 7:**
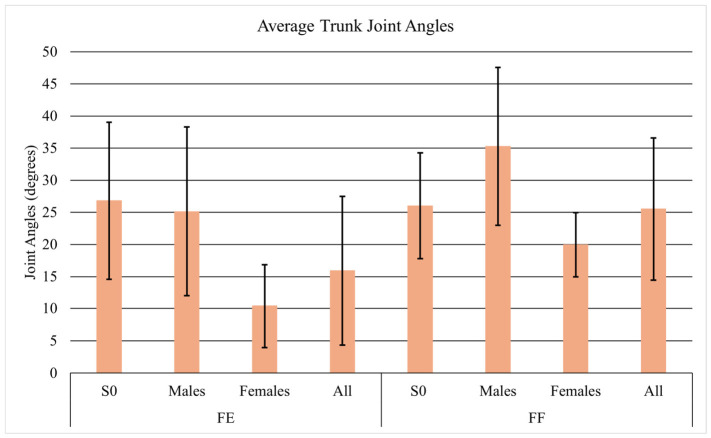
Average trunk angles with standard deviation for S0, males, females, and all participants.

Average joint angles differed between tasks. When facing each other, the subjects and S0 demonstrated hip angles of 106.0° and 78.9° (Figure 5), knee angles of 74.7° and 103.4° (Figure 6), and trunk angles of 15.9° and 26.8° (Figure 7), respectively. When facing forward, hip angles were 91.3° and 78.5° (Figure 5), knee angles were 83.0° and 101.3° (Figure 6), and trunk angles were similar at 25.5° and 26.0° (Figure 7).

### 3.3. Correlation

Table 1 shows the correlation values between the maximum lower back forces exerted by S0 and all subjects respectively and five different parts of the body, being the right and left knee, right and left hip, and trunk while performing each task. Similarly, correlation values were also found for the same joints and AP shear force.

For the hips, there was a positive medium correlation for S0 when he exerted the maximum lumbar forces in both tasks ranging from 0.27 to 0.53. The subjects experienced a negative medium correlation in the facing-each-other task ranging from −0.20 to −0.42. The correlation for the subject in the hips when facing forward was low.

In the knees, S0 experienced a medium negative correlation for both tasks ranging from −0.42 to −0.73. During these same tasks, the correlation for the subject was low and negative.

The correlation in the trunk for S0 showed a low negative correlation for both tasks. However, for the subject, the trunk had a medium positive correlation for both tasks ranging from 0.26 to 0.67.

### 3.4. A Linear Mixed-Effect Model

The results indicated that all data, including force and joint angle measurements, were normally distributed across participants. The summary table with a linear mixed-effect model, estimate value, 95% confidence intervals (CIs), *p*-values and effect sizes across tasks and between genders are listed in Table 2. Values less than 0.05 indicate significant differences.

The statistical significance of fixed effects was evaluated using *p*-values obtained from the linear mixed-effect models. Lifting task showed a significant effect on lower back forces and proximal joint kinematics (*p* < 0.05), indicating that the two lifting techniques produced different biomechanical outcomes. Gender also demonstrated significant effects for selected variables, particularly in lower back forces and trunk motion (*p* < 0.05). In contrast, the interaction between task and gender was not statistically significant for most variables (*p* > 0.05).

## 4. Discussion

This study utilized motion capture technology and an ergonomics tool to analyze the effects on the lower back when performing rescue tasks. Specifically, two motion-tracking systems were used simultaneously to collect data between two subjects performing a task where they are both facing forward and a task where they are both facing each other.

### 4.1. General Data Findings

The compressive forces were consistently higher than the AP shear forces in both tasks, which can be attributed to the study design, as patient lifting occurs primarily in the vertical direction with minimal lateral or rotational components [28]. Additionally, the average compressive force and AP shear force were significantly higher in S0 compared to the other subjects because S0 supported a larger portion of the mannequin’s upper-body load, whereas the lower body load was carried by the subjects. Considering that S0 also adopted a more upright and symmetric lifting posture with greater trunk-extensor muscle activation, this resulted in substantially higher compressive loads and increased shear forces than those observed in the other subjects [29].

The joint angles were also of significance because they tended to shift depending on which task was being performed. When facing each other, subjects demonstrated higher hip joint angles and lower knee joint angles compared to when they were both facing forward. This difference arises because facing each other changes the orientation of the torso, pelvis, and foot placement relative to the patient being lifted. To maintain balance, reach the object, and coordinate the lift, the subjects likely adjusted their joint positions: greater hip flexion and reduced knee flexion may have helped align the load path, distribute forces, and enhance stability [30].

When facing forward, the subjects needed to increase knee flexion, reduce hip flexion, and increase trunk flexion to reach the patient’s feet. During the leg lift, S0 showed lower hip joint angles and higher knee joint angles than the subjects, allowing S0 to keep a more neutral trunk while lifting the mannequin’s upper body. These findings suggest that in coordinated two-person lifts, the biomechanical roles of each individual influence overall movement patterns, affecting hip and knee joint angles in both S0 and the subjects [31].

### 4.2. Influence on Body Height, Body Weight, and Gender

The task-based differences also interact with individual physical characteristics. Body height and body weight are closely related biomechanical factors that influence how external loads are distributed across the spine. Taller individuals typically weigh more and have longer limbs, which increases spinal loading even when carrying the same weight [32]. Although both shear and compressive forces increase with height, body weight has a stronger influence when subjects adopt flexed postures [32,33]. As a result, taller and heavier individuals may face an increased risk of musculoskeletal disorders due to greater trunk and lower body loading [33].

In both tasks, male participants exhibited higher compressive and AP shear forces compared to female participants. The differences in forces may be due to the variations in lifting techniques, strength distribution, and body dimensions seen between genders [34,35]. Males tend to have longer limbs and a larger limb-to-torso ratio, which alters their center of gravity and loading patterns during lifting [34]. Female participants initiated lifts primarily with their knees, followed by the hips and trunk, whereas male participants relied more on the lower torso to carry most of the load.

When comparing the joint angles, it was found that females exhibit higher hip joint angles in both tasks compared to males. Females typically have a wider pelvis and larger pelvic tilt angle than males, which leads them to rely more on pelvic-driven lifting and results in greater hip flexion during lifts [36]. Unlike females, males generally have lower flexibility and a higher center of mass, which leads them to rely more on trunk-driven lifting. Consequently, males in this study exhibited greater trunk flexion and less hip flexion.

### 4.3. Interaction Effects Among Trunk, Knee, and Hip

During both tasks, the subjects showed a moderate correlation between trunk motion and both compressive and AP shear forces. These findings suggest that increased trunk involvement is associated with greater spinal loading, which is consistent with established biomechanical principles. However, S0 did not exhibit this correlation. This is likely because S0 was responsible for lifting the mannequin’s upper body, which carried a substantially heavier load than the legs.

However, the knees showed the opposite pattern, with a high correlation to AP shear forces and a moderate correlation to compressive forces in both tasks for S0. Because S0 was lifting the heavier load, he relied more on his knees than his hips to perform the task, reflecting a knee-dominant lifting strategy under higher load conditions. As a male participant, S0 also tended to use his knees as the primary contributor to the lift rather than the trunk and hips [36].

In both tasks, S0 demonstrated a medium positive correlation in the hips for both AP shear forces and compressive forces, whereas the subjects showed a medium negative correlation in the facing-each-other task. As noted earlier, the hips were likely not the primary contributors for S0, who appeared to rely more on his knees during the lift [36]. In contrast, the subjects, who were lifting the lighter and lower portion of the mannequin’s legs, relied more on their trunk flexion and consequently exhibited reduced hip flexion.

From a biological perspective, these findings help identify movement patterns that may contribute to increased mechanical stress on the lumbar spine, particularly under more demanding or repetitive conditions. The observed correlations do not directly translate to injury risk but instead indicate potential risk factors related to posture and coordination.

### 4.4. Difference Between Tasks

Both compressive and AP shear forces were higher for S0 and the subjects during the facing-each-other task compared to the facing-forward task. For S0, there was no significant difference in spinal forces between the two tasks, whereas the subjects exhibited an approximate 400 N difference. As previously noted, in two-person lifts, biomechanical roles shift depending on each person’s position, influencing joint angles, movement patterns, and the forces they exert on one another [31]. In the facing-each-other task, both S0 and the subjects lifted the mannequin from a squatting position with the weight positioned directly in front of them. In contrast, the facing-forward task mimics a deadlift, where the lower back remains straight and the leg muscles contribute more to the lift. This posture enables a straighter back, reducing hip flexion [34] and thereby lowering overall spinal loading [37]. However, to reach the mannequin’s legs in the facing-forward task, greater trunk flexion is required.

In both tasks, males experienced greater spinal loading than females. During the facing-forward task, both genders adopted similar lifting postures when raising the mannequin from the floor, except for trunk flexion, where males flexed approximately 15° more than females. It appears that males huddled their bodies more, resulting in higher spinal compressive and shear forces [38]. In the facing-each-other task, males also exhibited about 10° more trunk flexion than females. Considering the coordination between the trunk and hips, males showed roughly 20° less hip flexion compared to females, which aligns with findings from a previous study [39].

### 4.5. Suggestions

(1) Emphasize upright, neutral trunk postures when performing transfers: When lifting heavy weights, subjects have to engage their lower body strength by increasing knee flexion to support the load while keeping the upper body straight. Rescue workers should therefore be encouraged to minimize forward bending by maintaining a neutral spine and staying as close to the patient as possible during handling tasks. For relatively light weights, subjects should maintain upright and neutral trunk and hip postures. Prior studies indicate that increased trunk flexion is associated with elevated spinal loading and higher risk of low back injury, particularly when approaching end-range flexion (e.g., ~60°) [40]. Based on these findings, we suggest maintaining trunk flexion within approximately 20–30° during lifting tasks to avoid excessive loading on spinal tissues. In addition, lifting strategies that increase knee flexion (>60°) and moderate hip flexion (~45–70°) are consistent with squat-style lifting mechanics, which redistribute load from the lumbar spine to the lower extremities [37].
(2) Enhance movement coordination training, especially for mixed-gender teams: Notable differences in force patterns were observed between male and female subjects, with males generally generating higher compressive and AP shear forces in both tasks. Training programs should emphasize coordinated movement strategies to ensure synchronized timing and balanced distribution of effort, which would reduce the risk of sudden or uneven spinal loading.
(3) Perform two-person partner lifts with both people facing forward: This posture promotes a more neutral lower back position and reduces excessive spinal loading. Facing forward allows both people to better align their bodies with the load, improving force distribution and coordination. Although this study did not directly test standardized EMS protocols, the facing-forward technique resembles a squat lifting strategy, which is widely recommended in occupational ergonomics to reduce trunk flexion and improve load distribution [41]. Our findings are consistent with these principles. However, the current literature does not universally support one lifting technique as superior. Instead, different techniques redistribute spinal loads in different ways [37]. Therefore, our recommendations emphasize optimizing joint coordination and limiting excessive trunk flexion. Under the conditions of the present study, the facing-forward technique was associated with lower lumbar loading indicators; however, this finding requires further validation in more realistic EMS populations and more complex scenarios.

## 5. Conclusions

This study successfully employed motion capture systems in combination with a digital human modeling ergonomics tool to evaluate biomechanical risk factors associated with two-person rescue lifting techniques and their influence on lower back loading. Strong relationships were identified between lifting technique and spinal forces, with the facing-each-other task producing higher compressive and AP shear forces, increased hip flexion, and reduced knee flexion compared to the facing-forward task. Additionally, differences in body dimensions and gender were closely associated with variations in spinal loading. Trunk, knee, and hip motions also demonstrated significant correlations with lumbar forces. By simultaneously analyzing the interactions between two participants through dual motion-tracking systems, this approach offers deeper insights into coordinated lifting mechanics and supports the development of more effective musculoskeletal injury prevention strategies for emergency responders. Although this sample size was determined based on an a priori power analysis, a larger sample and the inclusion of both male and female participants would further enhance the generalizability of the findings. Additionally, incorporating real emergency transport scenarios would further strengthen their applicability.

## Figures and Tables

**Table 1:** Correlation values between force and the five joints. “FE” stands for the facing-each-other task and “FF” stands for the facing-forward task.

			Right Hip	Left Hip	Right Knee	Left Knee	Trunk
FE Task	S0 max	Compressive Force	0.38	0.35	−0.42	−0.53	−0.11
		AP Shear Force	0.37	0.27	−0.72	−0.73	−0.07
	Subject max	Compressive Force	−0.36	−0.42	−0.07	0.01	0.45
		AP Shear Force	−0.20	−0.27	−0.27	−0.24	0.26
FF Task	S0 max	Compressive Force	0.40	0.31	−0.43	−0.48	0.13
		AP Shear Force	0.53	0.47	−0.64	−0.69	−0.25
	Subject max	Compressive Force	−0.10	−0.04	0.06	0.05	0.67
		AP Shear Force	0.14	0.23	−0.16	−0.27	0.37

**Table 2:** Linear mixed-effect model results for lower back forces and joint angles.

Dependent Variable	Effect	β (Estimate)	95% CI	*p*-Values	Effect Size
Compressive Force (N)	Task (FF vs. FE)	−374.27	[−490.89, −257.65]	*p* < 0.001	0.65
	Gender (Male vs. Female)	697.54	[448.42, 946.78]	*p* < 0.001	
	Task × Gender	−40.00	[−239.34, 159.25]	*p* = 0.69	
AP Shear Force (N)	Task (FF vs. FE)	−124.09	[−167.85, −80.32]	*p* < 0.001	0.52
	Gender (Male vs. Female)	135.63	[58.43, 212.85]	*p* = 0.001	
	Task × Gender	−31.16	[−105.50, 43.14]	*p* = 0.40	
Trunk Angle (°)	Task (FF vs. FE)	9.31	[5.24, 13.37]	*p* < 0.001	0.53
	Gender (Male vs. Female)	15.14	[7.67, 22.61]	*p* < 0.001	
	Task × Gender	0.73	[−6.19, 7.64]	*p* = 0.83	
Right Hip Angle (°)	Task (FF vs. FE)	−20.87	[−31.05, −10.68]	*p* < 0.001	0.34
	Gender (Male vs. Female)	−22.62	[−36.70, −8.55]	*p* = 0.002	
	Task × Gender	15.25	[−1.84, 32.33]	*p* = 0.079	
Left Hip Angle (°)	Task (FF vs. FE)	−20.26	[−29.64, −10.87]	*p* < 0.001	0.29
	Gender (Male vs. Female)	−18.33	[−32.07, −4.59]	*p* = 0.01	
	Task × Gender	14.73	[−1.06, 30.52]	*p* = 0.067	
Right Knee Angle (°)	Task (FF vs. FE)	3.94	[−9.52, 17.40]	*p* = 0.56	0.07
	Gender (Male vs. Female)	−1.34	[−20.41, 17.73]	*p* = 0.89	
	Task × Gender	12.61	[−10.00, 35.23]	*p* = 0.27	
Left Knee Angle (°)	Task (FF vs. FE)	2.51	[−12.57, 17.59]	*p* = 0.74	0.05
	Gender (Male vs. Female)	−7.43	[−28.29, 13.43]	*p* = 0.48	
	Task × Gender	15.42	[−9.88, 40.72]	*p* = 0.23	

## Data Availability

The original contributions presented in this study are included in the article. Further inquiries can be directed to the corresponding author.
